# Limiting and optimal Strouhal numbers or tip speed ratios for cruising propulsion by fins, flukes, wings and propellers

**DOI:** 10.1098/rsif.2024.0730

**Published:** 2025-01-22

**Authors:** James R. Usherwood

**Affiliations:** ^1^ Structure and Motion Laboratory, The Royal Veterinary College, North Mymms, Hatfield AL9 7TA, UK

**Keywords:** Strouhal number, swimming, flapping, flight, efficiency, propeller

## Abstract

Swimming and flying animals produce thrust with oscillating fins, flukes or wings. The relationship between frequency *f*, amplitude *A* and forward velocity *U* can be described with a Strouhal number *St*, where *St* = 2*fA*/*U*, where animals are observed to cruise with St≈0.2–0.4. Under these conditions, thrust is produced economically and a reverse von Kármán wake is observed. However, propeller-driven craft produce thrust with steadily revolving blades and a helical wake. Here, the simplified aerodynamic geometry of lift-based thrust production is described, applicable to both oscillating and revolving foils. The same geometric principles apply in both cases: if the foil moves too slowly, it cannot produce thrust; if it moves too fast, it produces thrust with excessive power demand. Effective, economic thrust production by animals is not the result of oscillating foils or cyclic vortex shedding; rather, the selection of amplitude and frequency, and wake vortex structure, are corollaries of driving an efficient foil velocity with finite amplitudes. Observed Strouhal numbers for cruising animals appear too low for optimal mechanical efficiency; however, the deviation from optimal efficiency may be small, and there are physical and physiological advantages to relatively low amplitudes and frequencies for swimming and flapping flight.

## 1. Introduction

**Figure 1.**
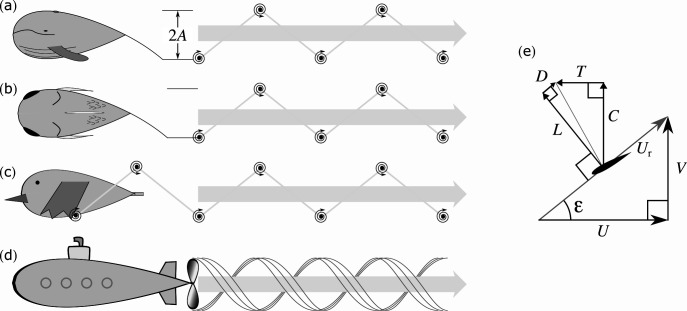
Fluke (*a*), fin (*b*), wing (*c*) and propeller (*d*) and a representative thrust-producing foil section (*e*) operating at *St* = 0.4 (𝜆 = 0.8) and (for the section) lift-to-drag ratio (LDR) = 6. The idealized wakes of swimming and flying animals produce thrust and a reverse von Kármán wake from the shed stopping/starting vortices; the trailing wing-tip vortices of the propeller produce a helical wake. The wake structures associated with body drag and (for the flier) lift are not included. The foil section (*e*)—its profile and angle—are for display purposes only. Symbols defined in text.

Swimming and flying animals have been observed to adopt a limited range of Strouhal numbers during broadly steady ‘cruising’ locomotion [1–3]. This is associated with a vortex wake of shed stopping and starting vortices and a net backward ‘thrust wake’ sometimes termed a ‘reverse von Kármán street’ ([4]; see [5, fig. 105] (figure 1)). Experimental and theoretical studies on a range of oscillating thrust-producers, from pitching and plunging plates [4,6] to flexible and more biorealistic foils [7–10], indicate that the observed Strouhal numbers are broadly efficient, and extreme deviation from observed Strouhal numbers results in negative thrust production or uneconomically high power demand. Various accounts for this relationship between Strouhal number and efficiency have been proposed, with focuses ranging from interactions between wake and foil (e.g. [1]) extending to ‘wake resonance’ applicable to flexible foils [11], to unsteady aerodynamics [12,13] and leading- and trailing-edge vortex development, to drag and scaling arguments [14]. Here, a simple formulation is developed to show the relationship between lift : drag ratio, Strouhal number and mechanical efficiency. It is sufficiently general to be applied to—and is in fact developed from—propeller theory, in which—as vortices are not shed cyclically—Strouhal number is not a relevant metric. Instead, ‘tip speed ratio’ is the most convenient analogue.

## 2. Theoretical development

Here, the thrust-producing foil is represented by a single section (figure 1*e*). For the fins and flukes, the section can be considered a simple ‘average’ chord. For the flapping wings and revolving propeller, it is assumed that the representative section is close to the wing-tip, as more distal sections operate at higher velocity and so produce higher forces, moments and power demands. The analysis (inspired largely by [15], which acknowledges [16]; see also [17]) considers only the mechanical efficiency of thrust production and uses simplifying two-dimensional geometry; the forces of weight support, and various induced flow velocities due to finite foil lengths, are not included.

Free-stream flow *U* and the relative velocity due to flapping *V* produce a resultant air velocity *U*_r_ at an angle ε to *U* (the complementary angle to ε is the ‘helix angle’ in propeller terminology). This geometry is determined by—or is an alternative description of—Strouhal number, tip speed ratio λ or advance ratio. Tip speed ratio is the ratio of tip speed (–) *V* to forward speed (–) *U* (advance ratio is its inverse). For a propeller, or a foil oscillating with triangular waveform,


(2.1) V=Uλ=4Af=2U St

(note that, Strouhal number relates to vortex spacing, so the foil travels 4*A* in period 1 */f*) and


(2.2) $$\varepsilon =\tan^{-1}\left(\text{V}/U\right)=\tan^{-1}\left(\lambda \right)=\tan^{-1}\left(2\;St\right).$$

Lift *L* acts perpendicular to the resultant air velocity *U*_r_; drag *D* of the foil acts in the direction of *U*_r_ (note that body drag, in the direction of *U*, is not considered, but would be the opposite of *T* in steady cruising). The relationship between drag and lift is given by the lift-to-drag ratio (LDR),


(2.3) $$D=\frac{L}{\text{LDR}}.$$

LDR is assumed to be a property of the foil (and its angle of incidence, assumed to be appropriate) and independent from *St* (or λ). Thrust *T* acts opposite to *U*, and the force *C* that demands torque and power acts perpendicular to *T*, in the direction of *V*. From the trigonometry of figure 1*e*,


(2.4) T=Lsin⁡(ϵ)−Dcos⁡(ϵ);

(2.5) C=Lcos⁡(ϵ)+Dsin⁡(ϵ).

The useful mechanical power supplied *P*_out_—in steady cruising, to overcome body drag—is


(2.6) $$P_{\text{out}}=TU.$$

The mechanical power demanded *P*_in_ due to the force *C* is


(2.7) $$P_{\text{in}}=CV.$$

The ratio of *P*_out_ to *P*_in_ is the mechanical efficiency η (note that induced flows are not considered—this is effectively a single blade element simplification without actuator disc corrections). Combining and simplifying the above equations gives η in terms of LDR and λ or *St*,


(2.8) $$\eta =\frac{TU}{CV}=-\frac{1-\lambda \;\text{LDR}}{\lambda \;\text{LDR}\;+\;\;\lambda^{2}\;}=-\frac{1-2\;\text{LDR}\;St}{2\;\text{LDR}\;St\;+\;4\;St^{2}\;}.$$

**Figure 2.**
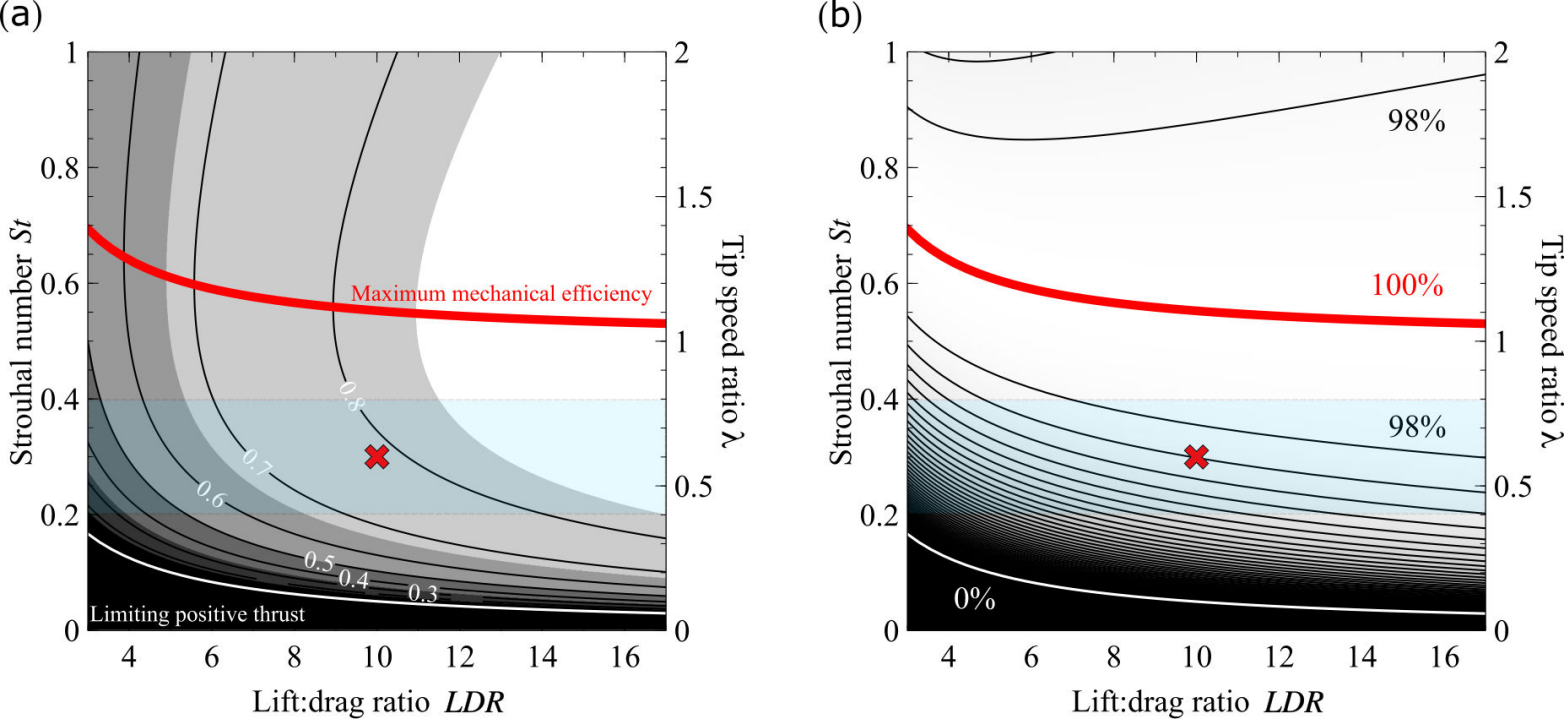
The surface (*a*) for mechanical efficiency as functions of lift : drag ratio and *St* or 𝜆. The same surface but normalized by the maximum efficiency at each LDR (*b*) emphasizes the rapid change in efficiency below the *St* = 0.2–0.4 region; contours show 2% changes. The blue-shaded region indicates the approximate range of observed LDR and *St* across swimming and flying animals. The red cross is at LDR = 10, *St* = 0.3 (𝜆 = 0.6), where 𝜂 = 0.786. The maximum efficiency for LDR = 10, 𝜂 = 0.819, requires *St* = 0.552 (𝜆 = 1.10). Operating at an intermediate biological *St* incurs a 4% reduction from maximal mechanical efficiency (for LDR = 10); however, maximum efficiency would require an increase in *St*—and so V—of 84%.

The resulting surface (figure 2) is displayed for *LDR* from 3 to 17, covering the range of maximum lift : drag ratios reported for isolated bird wings ([18]; see also [19]).

The optimum *St* or λ resulting in maximum efficiency, *St*_opt_ or $\lambda_{\text{opt}}$, as functions of LDR are found by solving dη/dSt=0 or dη/dλ=0, giving


(2.9a) $$\lambda_{\text{opt}}=\frac{1+\sqrt{1+{\text{LDR}}^{\;2}}}{\;\text{LDR}};$$

(2.9b) $$St_{\text{opt}}=\frac{1+\sqrt{1+{\text{LDR}}^{\;2}}}{2\;\text{LDR}}.$$

These tend to $\lambda_{\text{opt}}$ = 1 (*St*_opt_ = 0.5; i.e. ϵ = 45°) at high LDR. The limiting λ or *St*, $\lambda_{\text{lim}}$ or *St*_lim_, below which thrust cannot be produced, is (from equation 2.4):


(2.10a) $$\lambda_{\lim}=\frac{1}{\text{LDR}},$$

(2.10b) $$St_{\text{lim}}=\frac{1}{2\;\text{LDR}},$$

which is broadly consistent with a range of experimental and theoretical studies of oscillating foils [3,4,6,8–10,14].

## 3. Discussion and implications

Animals are observed to operate between *St* = 0.2 and *St* = 0.4, which is above the predicted limiting *St*, but rarely above those predicted to maximize mechanical efficiency ((2.9), figure 2). The reported range falls somewhat below *St*_opt_. Some model assumptions might bias the prediction high: e.g. deviation from triangular waveforms would result in periods of higher *V*. Other assumptions would bias low—e.g. the representative foil section is currently taken as the wing-tip, which is clearly extreme, and no account is taken of induced flows. There are numerous cases where the assumptions are particularly questionable, for instance eel-like swimming, or flight styles with very asymmetric or variable upstroke to downstroke ratios or with brief periods of gliding (consider doves and swallows). However, the observed range results in efficiencies very close to the optimal achievable for any given LDR (figure 2). Further, there are fluid-dynamic, mechanical and physiological pressures towards low amplitudes and frequencies. High-amplitude oscillations of fins and flukes would result in high ‘body’ drag; and high flap amplitudes impinge on the aerodynamic economy of weight support in flapping fliers as the average projected span decreases. Oscillating foils, especially wings, at high frequencies may be costly because of inertial power demands associated with accelerating the wing mass; and the brief contractions necessary for high-frequency oscillation increase the activated muscle mass demanded per joule of work supplied due to constraints to instantaneous muscle power [20]. The geometry developed here confirms an aerodynamic constraint prohibiting efficient—or indeed any—thrust production with wings flapping too slowly, and ties in with why small birds flap in a manner that compromises aerodynamic economy, presumably due to muscle constraints [21].

This analysis supports the notion that the mechanism underlying selection of *St* in animal swimmers and fliers is not the result of some interaction with the vortex wake (agreeing with [14]). Rather, it is a consequence of operating foils at suitable velocities for given lift : drag ratio and forward speed to achieve thrust and high (though perhaps not exactly optimal) efficiency—without including any foil-wake interactions. The ‘reverse von Kármán’ vortex wake may be considered an epiphenomenon or corollary of achieving effective λ with oscillations of finite amplitude. Neither the wake—nor, indeed, the oscillations—are the cause of efficient thrust production; suitable tip speed ratios are also achievable with steadily rotating propellers. Analogy is sometimes made between vortex wakes and footprints (e.g. [22]): both carry information regarding gaits, and possibly indicate something about gait mechanics. However, footprints are not fundamentally necessary: it is practically feasible to walk or run over a stiff substrate without leaving any footprints. Similarly, it is theoretically possible—with infinitely long foils and neglecting viscosity—to produce thrust with wings, fins or propeller blades without accelerating the fluid or leaving a wake. This is clearly a theoretical extreme, but it does demonstrate that a wake is not fundamental to the creation of thrust, just as the generation of footprints is not fundamental to the mechanics of walking or running.

The principles here also show that (as would be familiar to propeller designers), even with a perfectly and extremely twisted flapping or propeller foil, very inboard sections cannot contribute to thrust. If the tip operates at optimal efficiency, inboard sections operate at functionally lower speed ratio and suboptimal efficiency. In this case, locations inboard of $\lambda_{\text{lim}}/\lambda_{\text{opt}}$ times the radius (or single wing length) should be absent (or, more practically, minimized). In propeller terminology, sites inboard of this location would be ‘windmilling’ as opposed to thrust-producing. From expressions (2.9) and (2.10),


(3.1) $$\frac{\lambda {\;}_{\lim}}{\lambda {\;}_{\text{opt}}}=\frac{1}{1+\sqrt{1+{\text{LDR}}^{2}}}.$$

For LDR = 10, this results in 0.09; sections in the inboard 9% portion of the wing cannot produce thrust. This may provide one account for the stalked or ‘petiolated’ [23] wings typical of damselflies and craneflies: if they support weight with propeller-like wing motions, the inboard sections can only be detrimental and so should be minimized. This should be viewed as a tentative hypothesis; there may be many other advantages to such wing designs, and the propeller simplification, while generally suitable for thrust, does not apply to weight support in fast forward flapping flight.

The assumption made here is that there is value to considering a model where the LDR of the entire thrust-producing foil is represented by the geometries and aerodynamic characteristics of a single ‘representative’ foil section. This assumes that the section is always appropriately aligned to the flow—presumably to maximize LDR—implying an appropriate coupling between heaving and pitching of the section—meaning that this does not need to be modelled explicitly. While this ignores many factors known to influence whole-foil performance, especially planform and variation in section properties, it allows some of the consequences of these details to begin to be inferred. For instance, if high aspect ratio planforms generally have higher LDR, why do not all wings and fins have a high aspect ratio? Consider the changes in whole-foil design that might be appropriate if thrust at lower speeds was important, perhaps during a start or take-off. Low *U* would—all other things being equal—result in excessive *St* (λ), and a loss of mechanical efficiency if above the maximum efficiency condition of figure 1. One response to this might be to increase chord length (decrease aspect ratio) [24] and exploit high lift devices—the emarginate primaries of vultures, storks and pheasants being a prime candidate [25]—despite a slight detrimental decrease in LDR. As long as the decrease in LDR was not too extreme, the associated reduction in flap velocity *V* for a given thrust demand or power availability could reduce *St* (λ), returning the geometries towards high mechanical efficiency. Identifying some of the primary geometric principles behind economical thrust production, despite relying on a number of generalizations and approximations, provides not only a general account for broad kinematic relationships but also offers a starting point for considering more detailed aspects of form and function.

## 4. Conclusion

The geometry of fin, fluke or wing motion, and the various forces produced, explain conditions for effective and efficient thrust generation. Animals oscillate their foils sufficiently rapidly to generate thrust, and not faster than would be efficient; however, they may well operate somewhat below the perfectly optimal oscillation speed for their lift-to-drag ratio, resulting in a slightly suboptimal mechanical efficiency; this is presumably offset by one or more of a range of advantages to the considerably lower amplitudes and/or frequencies this permits.

## Data Availability

This article has no additional data.
